# 

**Published:** 1885-12-20

**Authors:** 


VOL. XV. DECEMBER 20, 1885. NO. 12

LIBRARY
INDEXED
S.G.O.

### THE SOUTHERN MEDICAL RECORD:

A MONTHLY JOURNAL OF PRACTICAL MEDICINE.

Terms: $2.00 per Annum, in Advance; 4 Copies $6.00; Single Copies 20 Cents.

*"QUICQUID PRÆCIPIES ESTO BREVIS."*

Address R. C. WORD, M.D., Managing Editor, Atlanta, Ga.

Entered at the Post-Office at Atlanta, as second-class matter.

